# Efficiency-Oriented Model Predictive Control: A Novel MPC Strategy to Optimize the Global Process Performance

**DOI:** 10.3390/s24175732

**Published:** 2024-09-03

**Authors:** Jiahong Xu

**Affiliations:** Robotics Institute, Ningbo University of Technology, Ningbo 315211, China; jiahongxu@nbut.edu.cn

**Keywords:** non-linear model predictive control, optimal control, optimization, intelligent system

## Abstract

Existing control strategies, such as Real-time Optimization (RTO), Dynamic Real-time Optimization (DRTO), and Economic Model Predictive Control (EMPC) cannot enable optimal operation and control behavior in an optimal fashion. This work proposes a novel control strategy, named the efficiency-oriented model predictive control (MPC), which can fully realize the potential of the optimization margin to improve the global process performance of the whole system. The ideas of optimization margin and optimization efficiency are first proposed to measure the superiority of the control strategy. Our new efficiency-oriented MPC innovatively uses a nested optimization structure to optimize the optimization margin directly online. To realize the computation, a Periodic Approximation technique is proposed, and an Efficiency-Oriented MPC Type I is constructed based on the Periodic Approximation. In order to alleviate the strict constraint of Efficiency-Oriented MPC Type I, the zone-control-based optimization concept is used to construct an Efficiency-Oriented MPC Type II. These two well-designed efficiency-oriented controllers were compared with other control strategies over a Continuous Stirred Tank Reactor (CSTR) application. The simulation results show that the proposed control strategy can generate superior closed-loop process performance, for example, and the Efficiency-Oriented MPC Type I can obtain 7.11% higher profits than those of other control strategies; the effectiveness of the efficiency-oriented MPC was, thereby, demonstrated.

## 1. Introduction

Systems and control are at the heart of many application domains, and the development of control strategies that enable intended behavior in an optimal fashion while satisfying constraints is one of the key benefits realized by the use of systems and control [1]. Devising control strategies to optimize a global objective directly, such as process performance, has also received increasing attention recently [2]. Within the context of the chemical process industry, for example, process performance usually refers to the process economics of the closed-loop process operations, which include a number of objectives: profitability, efficiency, variability, capacity, sustainability, etc. Optimal operation and the control of dynamic systems and processes is the ideal behavior leading to desirable process performance, and it has been an important subject of research for many years [3,4,5]. In view of the above, the aim of the devised control strategy is straightforward: to generate a closed-loop control sequence which can result in optimal global process performance.

**Figure 1:**
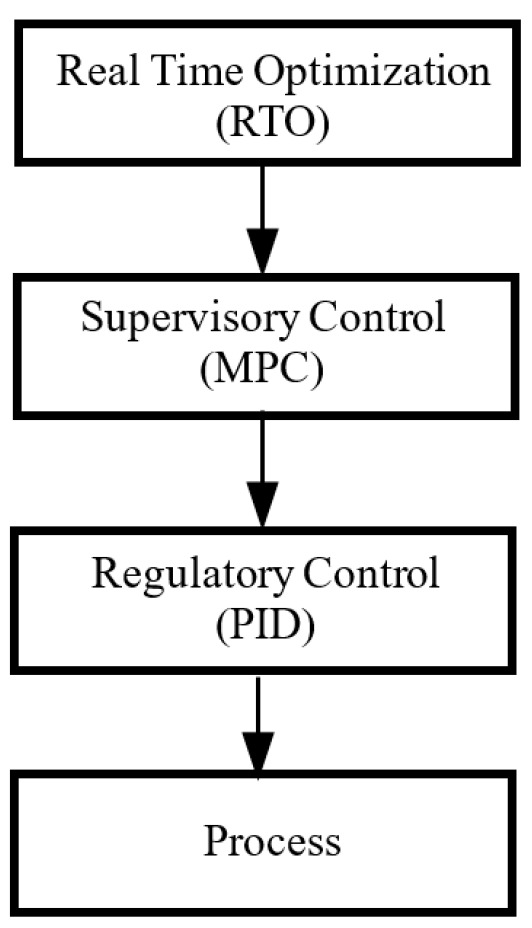
The multi-layer hierarchical architecture of RTO [11].

A cornerstone of the operation of chemical plants and processes to optimize the global process performance is calculating the optimum operating conditions and then maintaining them, despite the presence of measurement uncertainties and disturbances [6]. This kind of control strategy is typically categorized as real-time optimization (RTO), which can generate an acceptable global process performance. Specifically, the (economic) optimization and (tracking) control of plants are addressed in a multi-layer hierarchical architecture, as depicted in Figure 1. The problem of optimal operation and control has been divided into two issues: the upper RTO layer defines what the optimal operation is, and then the lower layer controls the process into this pre-determined optimal operation. This hierarchical RTO structure is popular in practical applications because it is easy to understand and simple to implement [7,8,9,10].

However, the RTO layer typically considers a simplified process performance which focuses solely on the steady process performance rather than the dynamic process performance, thus, it cannot enable the optimal operation and control in an optimal fashion. The supervisory control layer in Figure 1 is usually a form of model predictive control [12,13,14], which is an advanced control strategy developed for online optimal control problems, and which has been widely implemented in the chemical process industry [15]. The RTO-MPC structure can track the optimal steady state perfectly, but many practical problems require a paradigm that goes beyond steady state operation and embraces dynamic operation and online changing conditions [16].

In addition to the steady-state operation, there exist additional limitations in RTO strategy:

(1) The multi-layer hierarchical architecture introduces time-scale separation between different layers [17]. Specifically, the optimization of RTO relies on a steady-state model, and the plant must be sufficiently steady to reliably update the plant model [18]. However, the time-scale of the MPC layer is usually minutes, which is much faster than that of the RTO layer, and MPC will track sub-optimal setpoints between two RTO sample instants. In addition, detecting whether the plant is in the steady-state condition itself is not a simple task [19].

(2) There is a model mismatch between the RTO layer and the MPC layer. The optimization model used in the RTO layer is the non-linear steady-state model, while the MPC layer usually considers a simplified linear model [20]. This may result in an unreachable setpoint problem for the lower MPC layer [21].

(3) The RTO layer focuses solely on the economic performance of the steady operation, which may result in over-regulation behavior [22]. Specifically, the MPC layer mainly focuses on how well the MPC controls the process to maintain the optimal steady state, often neglecting the transient economic performance of the dynamic process.

(4) There is a growing need for a dynamic market-driven operation which requires a more efficient process operation [23], thus, a time-varying (dynamic) economic objective function should be considered. Since the hierarchical RTO structure is inherently a static steady state operation, it might be outdated in the next-generation manufacturing era.

In order to overcome the aforementioned issues, a novel control strategy for dynamic optimal operation and control is required, which should bridge the gap between optimization and control layers to enhance the global process performance.

The integration of the optimization layer and control layer can result in dynamic operations with better global process performances [24], thus, dynamic real-time optimization (DRTO) has been proposed, which uses a dynamic model in the RTO layer [25]. The optimization models used in the RTO layer and MPC layer are identical in DRTO, and the problem of model mismatch is avoided. Using a dynamic model in the optimization layer can lead to a dynamic operation whose process performance is better than that of a steady state operation. More advances in DRTO can be found in [26,27,28]. However, DRTO retains a two-layer structure and poses time-scale separation challenges.

Researchers recently proposed the Economic Model Predictive Control (EMPC) [29], which integrates the two layers in DRTO into one layer. Unlike tracking MPC (TMPC), which uses a quadratic objective function, EMPC incorporates a general cost function that directly accounts for process economics, and it enables a feedback-optimizing control [30]. EMPC enables a dynamic operation that results in optimal economic benefits generated by the process dynamics within the prediction horizon. However, since EMPC is typically a finite-horizon optimization, it may introduce recursive feasibility and closed-loop stability issues. Terminal constraints are usually used in EMPC to guarantee recursive feasibility and stability [31], but the optimality of the global process performance may deteriorate accordingly. EMPC without terminal constraints has been investigated recently, and the developments on this kind of EMPC depend significantly on dissipativity notions of optimal control problems, which can be found in [32,33,34]. The underlying motivation for this terminal-free EMPC is the so-called turnpike property (see [35]). However, the turnpike property and dissipativity both have strict requirements on the controlled plant.

To summarize, the existing control strategies like RTO, DRTO, and EMPC can optimize the global process performance to some extent, yet none of these realizes the optimal global process performance.

In making online optimal operation and control possible, smart sensors play an important role. Sensors gather real-time operational data to feedback to the control loop, and the accuracy of the data determines the performance of the control strategy [36]. Thanks to the rapid developments of smart sensors nowadays [37,38,39], intelligent control strategies with nested control structures can be devised because the delay within the control loop has been greatly alleviated. In essence, the connections between sensors and the MPC technique are similar to the relationships between private documents and the Large Language Model (LLM) technique in the Natural Language Processing (NLP) community which has a concept known as Retrieval-Augmented Generation (RAG) [40]. The MPC technique acts as a brain that makes decisions to achieve optimal performance, but the correctness of the policy depends on the feedback from the physical world (process operation) through smart sensors. The control strategy proposed in this manuscript assumes that the system states can be obtained ideally by the smart sensors, which means the information is obtained rapidly and accurately. This assumption places very high demands on smart sensors, and we will investigate the performance of the nested structure based on delayed information in the future.

The main contribution of this paper is to propose a novel control strategy that can enable optimal operation and control behavior in an optimal fashion. Specifically, (1) this paper proposes the concepts of optimization margin and optimization efficiency, and the control strategy which aims to optimize the optimization efficiency is denoted as the efficiency-oriented controller; (2) the key characteristic of the efficiency-oriented controller is a nested structure to optimize the global process performance; (3) two specific efficiency-oriented controllers, named the Efficiency-Oriented MPC Type I (EfiMPC1) and the Efficiency-Oriented MPC Type II (EfiMPC2), are proposed and discussed in detail. The MPC framework is used here because it is an industrially successful attempt to realize closed-loop optimal controls by a receding horizon application of open-loop optimal controls [41].

The framework of this paper is as follows. Section 2 outlines the preliminaries of the efficiency-oriented controller, especially the concepts of optimization efficiency, terminal truncation term, and the nested structure for optimizing the global process performance. Two types of efficiency-oriented controllers are constructed in Section 3. Section 4 presents the simulation results of the proposed efficiency-oriented controller over the CSTR application. Finally, Section 5 concludes the paper.

## 2. Preliminaries of Efficiency-Oriented MPC

### 2.1. Optimization Margin and Optimization Efficiency

Mathematically speaking, the optimizing control problem considering global process performance has the following form:

(P1) $$J_{P}^{*}=\underset{u_{P}}{\min}J_{P}\left(u_{P};x_{0}\right)=\underset{u_{P}}{\min}\int_{t_{0}}^{t_{f}}l_{P}(x\left(t\right),u_{P}(t))dt\;$$

$$s.t.\dot{x}=f(x\left(t\right),u_{P}(t)),x_{0}=x(t_{0})$$

$$x\left(t\right)\in X,u_{P}\left(t\right)\in U,t_{0}\leq t\leq t_{f}$$

where $x_{0}$ is the initial system state, $l_{P}$ is the general process performance with respect to the system states and the control inputs, f is the non-linear model of the system, and X and U are constraints of the system states and control inputs, respectively. $J_{P}$ is the global process performance function from the initial time instant $t_{0}$ to the final time instant $t_{f}$, $J_{P}^{*}$ is the optimal global process performance, and $u_{P}^{*}$ is the corresponding optimal input trajectory.

There are two issues here: (1) the optimal solution $u_{P}^{*}$ may be off-line sub-optimal in the closed-loop perspective considering disturbances and uncertainties; (2) the optimization horizon is so large that the optimization problem is hard to solve. Therefore, researchers favor using the receding horizon concept to approximate the problem (P1).

A receding horizon strategy tries to solve the following optimization problem at every sample instant $t_{k}$:

(P2) $$J_{k}^{*}=\underset{u_{k}}{\min}J_{k}(u_{k};x_{k})=\underset{u_{k}}{\min}\int_{t_{k}}^{t_{k}+H}l_{F}(x\left(t\right),u_{k}(t))dt+V_{F}(x_{k+H})$$

$$s.t.\dot{x}=f\left(x\left(t\right),u_{k}\left(t\right)\right),x_{k}=x\left(t_{k}\right),x_{k+H}=x\left(t_{k}+H\right)$$

$$x\left(t\right)\in X,u_{k}\left(t\right)\in U,t_{k}\leq t\leq t_{k}+H$$

$$x_{k+H}\in X_{F}$$

where $t_{k}$ for k=0, 1,… is the current sample instant, $x_{k}$ is the current system state, H is the finite prediction horizon, and $x_{k+H}$ is the terminal state at the end of the prediction. The stage cost of the controller, $l_{F}$, is usually a penalty of the tracking error with respect to the optimal setpoint $\left(x_{s},u_{s}\right)$, which is obtained from the RTO layer:

$$\left(x_{s},u_{s}\right)=\underset{x,u}{\mathrm{minimize}}l_{P}(x,u)$$

$$s.t.\dot{x}=f(x,u)$$

x∈X,u∈U

The obtained $\left(x_{s},u_{s}\right)$ relates the process performance metric $l_{P}$ with the control performance metric $l_{F}$, and the optimization problem (P2) only considers the steady process performance around $\left(x_{s},u_{s}\right)$.

Since problem (P2) only considers the local control performance from $t_{k}$ to $t_{k}+H$, the closed-loop stability of the system cannot be ensured. Devising the terminal cost $V_{F}$ and the terminal constraint $X_{F}$ off-line can guarantee the closed-loop stability, but the global process performance will deteriorate accordingly.

In problem (P2), $J_{k}$ is the open-loop local performance function, $J_{k}^{*}$ is the optimal local performance, and $u_{k}^{*}$ is the corresponding optimal solution. At every sample instant $t_{k}$, solve the problem (P2) repeatedly, and execute the first control action $u_{k}^{*}\left(t_{k}\right)$ into the plant. The resulting implicit closed-loop input profile has the following form:

$$u_{F}^{*}\left(t\right)=u_{k}^{*}\left(t_{k}\right),\;for\;t\in \left[t_{k},t_{k+1}\right),k=0,\;1,\ldots$$

The closed-loop system has a corresponding closed-loop process performance, which has the following form:

$$J_{F}^{*}=\int_{t_{0}}^{t_{f}}l_{P}\left(x\left(t\right),u_{F}^{*}\left(t\right)\right)dt$$

There is a problem: what is the optimality of $J_{F}^{*}$ compared with $J_{P}^{*}$? Since problem (P2) lacks the ability to optimize $J_{F}^{*}$, the closed-loop process performance is merely a by-product of the control performance. Thus, problem (P2) is an optimization based on the control performance with respect to $\left(x_{s},u_{s}\right)$, and problem (P2) lacks feedback to the operational layer to optimize the global process performance online directly.

The introduction of the terminal cost $V_{F}$ will also deteriorate the global process performance [42]. Each pair of $\left(V_{F},X_{F}\right)$ can be regarded as a specific control strategy, and if the closed-loop solution $u_{F}^{*}$ is not desirable compared with the ideal solution $u_{P}^{*}$, the corresponding closed-loop process performance $J_{F}^{*}$ will suffer.

It is necessary to modify $\left(V_{F},X_{F}\right)$ to enable them to optimize the global process performance at the process control layer online. Unlike traditional $V_{F}$, which only penalizes control performance, we propose in this paper a new terminal truncation term $V_{ter}$ which can optimize the global process performance, and $X_{ter}$ represents the corresponding terminal constraint. Evaluating the global process performance is equivalent to evaluating the optimality of the control strategy itself, resulting in the following finite-horizon optimization problem:

(P3) $$J_{C,k}^{*}=\underset{u_{H},u_{q}}{\min}J_{C,k}=\underset{u_{H},u_{q}}{\min}\int_{t_{k}}^{t_{k}+H}l_{P}(x\left(t\right),u_{H}(t))dt+V_{ter}(u_{q};x_{k+H})$$

$$s.t.\;\;\;\dot{x}=f\left(x\left(t\right),u_{H}\left(t\right)\right),x_{k+H}=x\left(t_{k}+H\right)$$

$$x\left(t\right)\in X,u_{H}\left(t\right)\in U,t_{k}\leq t\leq t_{k}+H$$

$$x_{k+H}\in X_{ter}$$

$$u_{q}=\underset{u_{q}^{\prime }}{\mathrm{argmin}}V_{ter}(u_{q}^{\prime };x_{k+H})\;$$

$$s.t.\;\;\dot{x}=f\left(x\left(t\right),u_{q}^{\prime }\left(t\right)\right)$$

$$x\left(t\right)\in X,u_{q}^{\prime }\left(t\right)\in U,t_{k}+H\leq t\leq t_{k}+H+q$$

where $u_{H}$ is the decision variable of the local process performance optimization, and $u_{q}$ is the decision variable of the terminal truncation term. $J_{C,k}$ is the measurement of the global process performance, and $J_{C,k}^{*}$ is the optimal global process performance. $X_{ter}$ can be constructed as a subset containing an equilibrium point to guarantee stability, and $V_{ter}$ is a function determined by the varying parameter $x_{k+H}$ and the decision variable $u_{q}$.

The function of $V_{ter}$ is to measure the global process performance based on the optimized terminal state $x_{k+H}$. The parameter q determines the size of $u_{q}$, and it can be considered as the optimization horizon of the terminal truncation term $V_{ter}$.

At every sample instant $t_{k}$, solve problem (P3) repeatedly and execute the first control input $u_{H}^{*}(t_{k})$ into the plant. The resulting implicit closed-loop input profile $u_{C}^{*}$ has the following form:

$$u_{C}^{*}\left(t\right)=u_{H}^{*}\left(t_{k}\right),\;for\;t\in \left[t_{k},t_{k+1}\right),k=0,\;1,\ldots$$

The closed-loop system has a corresponding closed-loop process performance:

$$J_{C}^{*}=\int_{t_{0}}^{t_{f}}l_{P}\left(x\left(t\right),u_{C}^{*}\left(t\right)\right)dt,$$

where $J_{C}^{*}$ is now the optimized closed-loop process performance.

**Figure 2:**
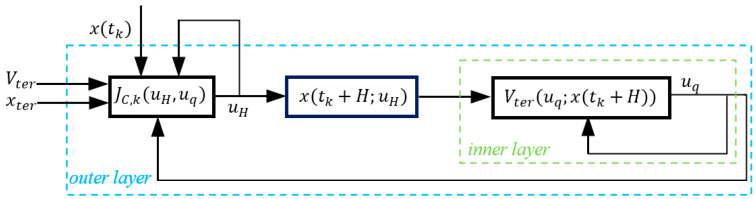
The nested structure of the efficiency-oriented MPC.

Problem (P3) represents the efficiency-oriented model predictive control discussed in this contribution, and it is intrinsically a nested optimization problem [43] where the inner layer optimizes the global process performance defined by the operational layer, and the outer layer optimizes the optimality of the local process performance. This nested structure is depicted briefly in Figure 2.

As shown in Figure 2, the optimal solution is affected by $V_{ter}$, thus, for each specific $V_{ter}$, it satisfies $J_{C}^{*}\left(V_{ter}\right)\geq J_{P}^{*}$. The aim of the efficiency-oriented MPC is to optimize the control strategy itself from the global process performance perspective, which means optimizing $J_{C}^{*}\left(V_{ter}\right)$ to approximate the ideal global process performance $J_{P}^{*}$. Due to the limitations of the optimization solver embedded in the controller, it may be unable to find the global optimal solution, and the practical solution obtained by the controller will result in a sub-optimal closed-loop process performance $J_{C}^{O}$. To further clarify the aim of the efficiency-oriented MPC, define the concepts of optimization margin and optimization efficiency based on $J_{P}^{*}$, $J_{C}^{*}$ and $J_{C}^{O}$ as follows.

**Definition** **1.** *For the efficiency-oriented MPC problem (P3), let* $J_{C}^{*}$ *denote the optimal closed-loop process performance. For the ideal global process of performance-optimizing control problem (P1), let *$J_{P}^{*}$ denote the ideal optimal global process performance. Define the **optimization margin** (OM) as follows:

$$OM=\frac{e^{-J_{C}^{*}}}{e^{-J_{P}^{*}}}\in \left[0,1\right].$$

Additionally, let $J_{C}^{O}$ denote the practical optimal closed-loop process performance obtained by a specific optimization solver in (P3). Define the ***optimization efficiency*** (OE) as follows:

$$OE=\frac{e^{-J_{C}^{O}}}{e^{-J_{P}^{*}}}\in \left[0,1\right].$$

The efficiency-oriented MPC aims to optimize both OM and OE, where OM=OE=1 indicates the optimal operation and control of the controlled system. A larger OM implies a better global process performance achieved by efficiency-oriented MPC ideally, while a larger OE implies a better practical process performance based on a specific solver.

**Figure 3:**
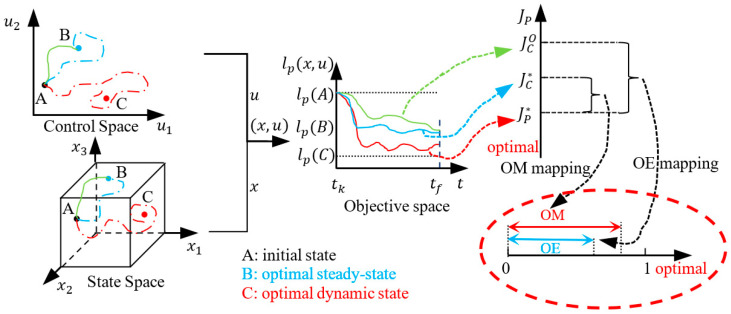
The brief ideas of optimization margin and optimization efficiency.

The concepts of OM and OE are depicted in Figure 3, where point A represents the initial state of the system in both the state space and decision space, point B denotes the optimal equilibrium point, and point C signifies the optimal dynamic point. The blue curve illustrates the optimal trajectory determined by the efficiency-oriented MPC, while the green curve portrays the practical trajectory determined by an embedded optimization solver. Additionally, the red curve showcases the ideal global optimal trajectory. By mapping the states and inputs of these three curves to the process performance $l_{P}$, we obtain three types of closed-loop process performance $J_{C}^{*}$, $J_{C}^{O}$, and $J_{P}^{*}$, satisfying $J_{P}^{*}\leq J_{C}^{*}\leq J_{C}^{O}$. Subsequently, OM and OE mappings transform $J_{C}^{*}$ and $J_{C}^{O}$ into a scalar range [0,1]. The problem of efficiency-oriented MPC is then reformulated as the optimization of OM and OE. In this study, we assume that the embedded optimization solver in efficiency-oriented MPC is ideal, always capable of finding the global optimal solution. Therefore, for simplicity’s sake, OM and OE are considered identical.

### 2.2. The Relationship between the Global Process Performance and the Terminal Truncation Term

**Figure 4:**
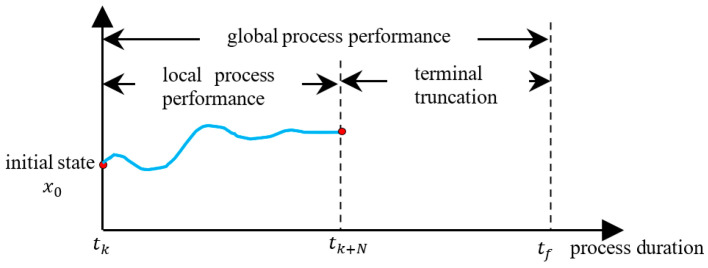
The concepts of global process performance and the terminal truncation term.

Optimal operation and control is the behavior controlled by an ideal control strategy, resulting in optimal closed-loop process performance. The open-loop process performance of the plant during the entire process operation is the global objective to be optimized online, and the corresponding optimal solutions comprise the closed-loop process performance. In this contribution, denote the open-loop process performance as *global process performance*, and divide it into two parts: (1) *local process performance*, and (2) *terminal truncation term*. The concepts of global process performance, local process performance, and terminal truncation term are illustrated in Figure 4.

$t_{k}$ represents the current sample instant, $t_{f}$ represents the final time of the process operation, $t_{k+N}$ denotes the last instant of the online optimization, and N represents the prediction horizon. Since $N\ll t_{f}-t_{k}$, a discrepancy exists between the process performance evaluated by the finite-horizon optimization problem and the global process performance. Denote the process performance from $t_{k}$ to $t_{k+N}$ as local process performance, and denote the value from $t_{k+N}$ to $t_{f}$ as terminal truncation term (or terminal truncation).

Ideally, a control strategy should optimize the global process performance from $t_{k}$ to $t_{f}$. However, only short horizon-based local process performance is optimized online. Let the optimization horizon for both TMPC and EMPC be N, indicating that they optimize local rather than global process performance. The objective function of TMPC typically focuses on minimizing the deviation from the optimal setpoints, whereas the objective function of EMPC can directly reflect the local process performance, thus, EMPC can achieve superior local process performance compared to TMPC.

An equality constraint is assumed for both TMPC and EMPC, ensuring that the terminal state $x(t_{k+N})$ aligns with the optimal steady state. Consequently, the terminal truncation terms for both TMPC and EMPC become identical constants, and superiority in local process performance implies superiority in global process performance, thus, the equality constraint functions as a terminal truncation technique, linking local and global process performance.

Although the EMPC framework discussed above can outperform TMPC, the terminal equality constraint employed in EMPC has the following drawbacks: (1) it represents a stringent constraint, reducing the feasible region and potentially limiting the optimization margin; (2) solving the online optimization problem becomes challenging; and (3) no optimization occurs within the terminal truncation term.

**Figure 5:**
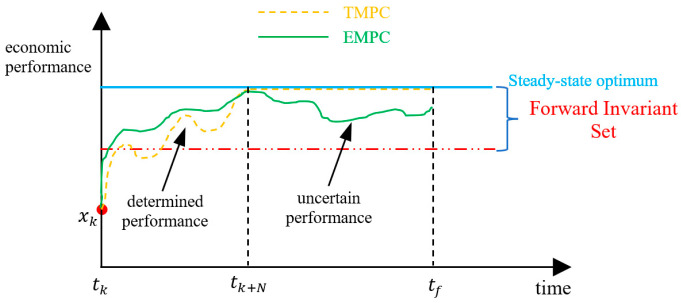
The process performance under terminal region constraint.

The terminal region constraint, denoted as $X_{f}\subset X$, represents another common type of terminal truncation technique. It can expand the feasible region, and $X_{f}$ is a forward invariant subset of the feasible state space X. However, this approach introduces additional challenges: (1) the terminal truncation term is hard to express numerically, and (2) the economic optimization is not the primary goal of the terminal truncation term. The primary role of the terminal region constraint is to ensure recursive feasibility and closed-loop stability, while the optimality of global process performance is often overlooked. As depicted in Figure 5, the red chain-dotted line, along with the blue optimal steady-state line, delineates a forward invariant region $X_{f}$. While the local process performance of terminal region-based EMPC exceeds that of TMPC, the process performance of the terminal truncation remains uncertain. Therefore, it is not accurate to conclude definitively that the global process performance of EMPC surpasses that of TMPC.

Despite the uncertainty of the terminal truncation, a terminal region constraint has the potential to achieve better process performance than a terminal equality constraint because the terminal equality constraint is a special case of the terminal region constraint. The challenge lies in guaranteeing the performance of the terminal truncation given a specific local process performance.

Therefore, an ideal control strategy should improve both the local process performance and the performance of the terminal truncation, which is the main motivation of the efficiency-oriented MPC proposed in this paper.

**Figure 6:**
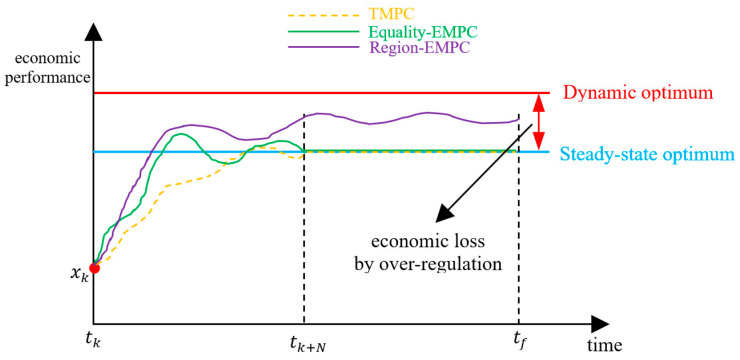
The over-regulation behavior.

The previous discussion assumes that the optimal solution is an equilibrium point, but in general the process performance function may not be positive or definite with respect to the optimal steady-state. Given a general process performance, a terminal truncation based on a steady-state can result in over-regulation behavior. Considering terminal equality-based EMPC as an example, the equality constraint forces the state towards the steady-state optimum, resulting in an economic loss. As illustrated in Figure 6, the local process performance of Equality-EMPC exceeds that of TMPC between $\left[t_{k},t_{k+N}\right]$, and the process performance of the terminal truncation is identical for both strategies. Consequently, the global process performance of Equality-EMPC is superior to that of TMPC. However, since there is a better dynamic optimal state (denoted by the red line), stabilizing the system at the steady-state optimum reduces the optimization margin of the controller. The difference between the dynamic optimum (red line) and the steady-state optimum (blue line) is the economic loss, denoted as over-regulation behavior.

Although the terminal region-based EMPC exhibits greater optimization potential than the terminal equality-based EMPC, as illustrated by Figure 6, a limitation of terminal region-based EMPC is the absence of optimization capability within the terminal truncation term. Efficiency-oriented MPC can release the potential of the optimization margin of the system under a generalized objective function by directly optimizing the terminal truncation term online to achieve superior global process performance.

Based on the preceding discussion, optimization efficiency (OE) can be calculated as:

$$OE=\frac{e^{-(V_{lpp}+V_{ter})}}{e^{-V_{TG}}}$$

where $V_{lpp}$ denotes the local process performance (with minimum values being optimal for $V_{lpp}$, $V_{ter}$, and $V_{TG}$), $V_{ter}$ denotes the terminal truncation term, and $V_{TG}$ denotes the ideal optimal global process performance. $V_{TG}$ is determined by the controlled system itself, and controllers can improve optimization efficiency by optimizing the value of the online process performance, denoted as $V_{CP}≔V_{lpp}+V_{ter}$. In the ideal situation, $V_{CP}$ equals $V_{TG}$ and OE=100%, otherwise, 0<OE<1.

A new type of controller is required that can optimize $V_{lpp}$ and $V_{ter}$ simultaneously to achieve improved optimization efficiency. We denote this novel controller as the efficiency-oriented (MPC) controller. The term “efficiency-oriented” signifies that the controller optimizes the system’s optimization efficiency effectively, with no (or little) wastage of optimization potential regarding the global process performance.

### 2.3. Understand Optimization Efficiency by Standard Optimal Control and MPC

The standard optimal control (SOC) problem $P_{\infty }(x_{0})$, which aims to solve to achieve optimal global process performance, has the following form [44]:

$$P_{\infty }\left(x_{0}\right):\;\underset{u\left(i,x_{0}\right)\in U}{\min}J_{\infty }\left(u,x_{0}\right)=\sum_{i=0}^{\infty }q_{\infty }\left(x\left(i,x_{0}\right),u\left(i,x_{0}\right)\right)$$

$$s.t.x\left(i+1,x_{0}\right)=f_{d}\left(x\left(i,x_{0}\right),u\left(i,x_{0}\right)\right),i=0,1,\ldots$$

$$x\left(i+1,x_{0}\right)\in X,u\left(i,x_{0}\right)\in U,i=0,1,\ldots$$

where $X\subseteq R^{n}$ and $U\subseteq R^{m}$ are constraints on system states and control inputs, respectively, $x\left(0,x_{0}\right)=x_{0}$ is the initial state, and $q_{\infty }(x,u)$ is the stage cost function.

$J_{\infty }\left(u,x_{0}\right)$ corresponds to the global process performance discussed in the previous subsection. Since a direct solution to $P_{\infty }(x_{0})$ is difficult to obtain, online solutions are preferred to approximate SOC problem. The finite horizon MPC framework is an excellent approximation, denoted as $P_{N}\left(x_{0}\right)$:

$$P_{N}\left(x_{0}\right):\underset{u\left(i,x_{0}\right)\in U}{\min}J_{N}\left(u,x_{0}\right)=\sum_{i=0}^{N-1}q_{N}\left(x\left(i,x_{0}\right),u\left(i,x_{0}\right)\right)+F(x(N,x_{0}))$$

$$s.t.x\left(i+1,x_{0}\right)=f_{d}\left(x\left(i,x_{0}\right),u\left(i,x_{0}\right)\right),i=0,\;1,\ldots ,N-1$$

$$x\left(i+1,x_{0}\right)\in X,u\left(i,x_{0}\right)\in U,i=0,\;1,\ldots ,N-1$$

where N is the prediction horizon, $X_{f}\subseteq R^{n}$ is a terminal region constraint, and $F:R^{n}\mapsto R$ is a terminal cost.

When people discuss “optimal control” within a finite horizon MPC framework, the term implicitly refers to the optimal solution of the simplified problem $P_{N}\left(x_{0}\right)$. Since $P_{N}\left(x_{0}\right)$ is merely an approximation of the original problem $P_{\infty }(x_{0})$, the so-called “optimal control” is actually “sub-optimal control” relative to $P_{\infty }(x_{0})$. Denote $P_{\infty }(x_{0})$ as the “superordinate objective” and $P_{N}\left(x_{0}\right)$ as the “subordinate objective”.

One critical aspect often overlooked is the degree of approximation of $P_{N}\left(x_{0}\right)$. As noted in [45], the objective in synthesizing a control structure is “to translate the economic objectives into process control objectives”. An efficiency-oriented controller aims to optimize these “translated control objectives” with regard to the global process performance.

$J_{\infty }\left(u,x_{0}\right)$ can be regarded as the global process performance $V_{TG}$, and $J_{N}\left(u,x_{0}\right)$ can be considered as the online process performance $V_{CP}$. The optimization efficiency can then be calculated as follows:

(1) $$OE=\frac{e^{-J_{N}^{*}\left(x_{0}\right)}}{e^{{-J}_{\infty }^{*}\left(x_{0}\right)}}=\frac{e^{-\left(V_{lpp}+V_{ter}\right)}}{e^{-V_{TG}}}$$

### 2.4. Classes of Process Systems

A continuous time-invariant non-linear system model is considered in this paper:

$$\dot{x}\left(t\right)=f\left(x\left(t\right),u\left(t\right),w\left(t\right)\right)$$

$$s.t.h\left(x\left(t\right),u\left(t\right)\right)=0\;\;\;\;\;\;\;\;$$

$$\;\;\;\;\;\;\;\;g\left(x\left(t\right),u\left(t\right)\right)\leq 0$$

where $x\in X\subseteq R^{n_{x}}$ is the state vector, $u\in U\subset R^{n_{u}}$ is the manipulated input vector, $w\in W\subset R^{n_{w}}$ is the disturbance vector, and $n_{x},n_{u}$ and $n_{w}$ are the dimensions of the state, input, and disturbance vectors, respectively. The input vector u is bounded in the subset U and satisfies the equality constraints $h\left(\cdot \right)$ and the inequality constraints g(·). X represents the set of admissible states. The disturbance vector is assumed that W={0} in this paper.

The discrete-time representation of this system is:

$$x(k+1)=f_{d}\left(x\left(k\right),u\left(k\right),w\left(k\right)\right)$$

where $f_{d}$ is discretized from f.

For the process systems of interest, consider a generalized objective function:

(2) $$l_{e}(x,u):R^{n_{x}}\times R^{n_{u}}\to R$$

which serves as a measure of the instantaneous process performance, and the accumulated value from the current sample instant $t_{k}$ to the final instant $t_{f}$ defines the global process performance $L_{pp}(t_{k},t_{f})$, which represents the superordinate objective:

$$L_{pp}\left(t_{k},t_{f}\right)=\int_{t_{k}}^{t_{f}}l_{e}\left(x\left(t\right),u\left(t\right)\right)dt=\int_{t_{k}}^{\infty }l_{e}\left(x\left(t\right),u\left(t\right)\right)dt=P_{\infty }\left(x_{k}\right)$$

However, the online optimization of $L_{pp}(t_{k},t_{f})$ is impractical due to the large prediction horizon $N=t_{f}-t_{k}$. MPC uses a finite horizon $N\ll t_{f}-t_{k}$ to construct a subordinate objective $L_{pp}\left(t_{k},t_{k+N}\right)$ to approximate $L_{pp}(t_{k},t_{f})$. Given Equation (1), the efficiency-oriented MPC aims to optimize $L_{pp}\left(t_{k},t_{k+N}\right)$ to best approximate $L_{pp}(t_{k},t_{f})$, thereby optimizing the global process performance online directly.

## 3. Efficiency-Oriented Model Predictive Control Algorithm

### 3.1. Periodic Approximation Technique

Let $\tau_{k}$ denote the current process sampling instant, and $t_{f}$ denote the final time of the process operation. The prediction horizon $H_{p}$ and the control horizon $H_{c}$ of the controller satisfy $H_{p}=H_{c}=N$, where $t_{k+N}=\tau_{k}+N\cdot \Delta t_{sam}$ is the end of the prediction, and $\Delta t_{sam}$ is the sampling interval, and $H_{ter}=max(0,t_{f}-t_{k+N})$ is the non-negative terminal horizon of the terminal truncation term $V_{ter}$. The current state of the system is $x_{k}=x\left(\tau_{k}\right)=x(t_{k})$, and $x_{k+N}=x(t_{k+N})$ is the terminal state at the end of the prediction horizon.

**Definition** **2.** *Assume there exists a periodic control sequence* $U_{q}=\left\{\left\{u_{q}\left(0\right),u_{q}\left(1\right),\ldots ,u_{q}\left(q-1\right)\right\}|u_{q}\left(j\right)\in U\subseteq R^{m},j=0,\;1,\ldots ,q-1\right\}$*, where *q≥1* is the period length. Define a mapping* $PA\left(\cdot ,\cdot \right):R^{n}\times R^{m\times q}\mapsto R$ *called Periodic Approximation, such that the terminal truncation term satisfies* $V_{ter}=H_{ter}\cdot PA$ *, where* PA *satisfies:*

(3) $$PA\left(x_{k+N},U_{q}\right)=\frac{\sum_{i=0}^{q-1}l_{e}(x_{q}\left(i\right),u_{q}(i))}{q}$$

$$s.t.x_{q}\left(0\right)=x_{k+N}$$

$$\;\;\;\;x_{q}\left(q\right)=x_{q}\left(0\right)$$

$$\;\;\;\;\;\;\;\;x_{q}\left(i+1\right)=f_{d}\left(x_{q}(i),u_{q}(i),0\right),i=0,\;1,\ldots ,q-1$$

**Figure 7:**
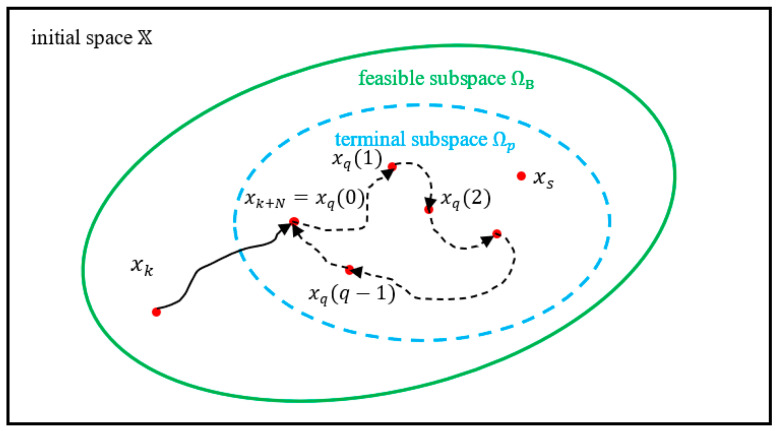
Periodic approximation technique for terminal truncation.

In Definition 2, $f_{d}$ is a discrete non-linear system, and $U_{q}$ is a feasible periodic control sequence with a period length of q. During the terminal horizon $H_{ter}$, the periodic control $U_{q}$ is implemented repeatedly, and the system performs a periodic operation $X_{q}=\{x\left(0\right),x\left(1\right),\ldots ,x(q-1)\}$, as illustrated in Figure 7.

**Remark** **1.** *There always exist feasible subsets* $\Omega_{p}$ *containing the optimal steady state* $x_{s}$ *in their interior to enablepPeriodic approximation, as a steady-state operation is a special kind of periodic approximation where* q=1*. The* $\Omega_{p}$ *is a forward-invariant set that can be considered the terminal region as defined in EMPC. In contrast, with the help of periodic approximation, the numerical value of the terminal truncation term becomes calculable*.

To improve the optimization efficiency, the controller should have the ability to find the optimal periodic approximation $PA^{*}$, leading to the better terminal truncation term $V_{ter}$ and the better local process performance $V_{lpp}$. $V_{lpp}$ and PA are connected by the terminal state $x_{k+N}$: $x_{k+N}$ is the optimization result of $V_{lpp}$ at the outer layer and then becomes the parameter of the PA at the inner layer.

### 3.2. Efficiency-Oriented MPC Type I

Denote the efficiency-oriented MPC based on the periodic approximation as the Efficiency-oriented MPC Type I (EfiMPC1). Its definition is as follows:

(E1) $$L_{E1}^{*}=\underset{U_{N},U_{q}}{\min}V_{lpp}^{E1}(x\left(t_{k}\right),U_{N})+V_{ter}^{E1}(x\left(t_{k+N}\right),U_{q})$$

$$s.t.V_{lpp}^{E1}=\int_{t_{k}}^{t_{k+N}}l_{e}\left(x\left(t\right),u\left(t\right)\right)dt$$

$$V_{ter}^{E1}=PA\left(x\left(t_{k+N}\right),u_{q}^{*}\right)\cdot H_{ter}$$

$$x\left(t_{k}\right)=x_{m}\left(\tau_{k}\right),\Delta t_{k}=t_{k+1}-t_{k}=\Delta t_{samp}$$

$$\dot{x}\left(t\right)=f\left(x\left(t\right),u\left(t\right),0\right),t_{k}\leq t\leq t_{k+N}$$

$$x\left(t\right)\in X_{U}\subseteq R^{n},\;u\left(t\right)\in U_{U}\subseteq R^{m},t_{k}\leq t\leq t_{k+N}$$

$$U_{N}=\left\{u_{N}\left(t_{k}\right),\ldots ,u_{N}\left(t_{k+N-1}\right)\right\}$$

$$u\left(t\right)=u_{N}\left(t_{k+i}\right),t_{k+i}\leq t<t_{k+i+1},i=0,\ldots ,N-1$$

$$U_{q}=u_{q}^{*}=\left\{u_{q}^{*}\left(0\right),\ldots ,u_{q}^{*}\left(q-1\right)\right\}$$

$$u_{q}^{*}=\underset{u_{q}}{\mathrm{argmin}}PA\left(x\left(t_{k+N}\right),u_{q}\right)$$

$$s.t.\;PA\left(x\left(t_{k+N}\right),u_{q}\right)=\frac{\sum_{j=0}^{q-1}l_{e}\left(x_{q}\left(j\right),u_{q}\left(j\right)\right)}{q}$$

$$x_{q}\left(0\right)=x\left(t_{k+N}\right),x_{q}\left(q\right)=x_{q}\left(0\right)$$

$$x_{q}\left(j+1\right)=f_{d}\left(x_{q}\left(j\right),u_{q}\left(j\right),0\right),j=0,\;1,\ldots ,q-1$$

$$x_{q}\left(j\right)\in X_{L}\subseteq R^{n},\;u_{q}\left(j\right)\in U_{L}\subseteq R^{n},j=0,\;1,\ldots ,q-1$$

In Equation (E1), $f_{d}$ is the discrete form of the continuous process system $f\left(x\left(t\right),u\left(t\right),0\right)$, $l_{e}$ is the generalized process performance function defined in Equation (2), $\Delta t_{k}$ is the sampling interval of the outer layer and inner layer optimization, $x_{m}\left(\tau_{k}\right)$ is the measurement of the current state. $X_{U}$ and $U_{U}$ are the constraints of states and control sequence in the outer layer, $X_{L}$ and $U_{L}$ are the constraints in the inner layer, $U_{N}$ and $U_{q}$ are the decision variables of the outer layer and the inner layer, respectively. $x\left(t_{k+N}\right)$ is the terminal state of the outer layer optimization, which then becomes the parameter of the inner layer optimization in PA.

At every sampling instant $\tau_{k},k=0,\;1,2,\ldots$, EfiMPC1 is solved online repeatedly, and the first control action of the solution $u_{E1}^{*}\left(0|\tau_{k}\right)=U_{N}\left(0\right)=u_{N}(t_{k})$ is implemented in the system. Based on the receding horizon strategy, the closed-loop control law obtained by EfiMPC1 is:

$$u_{E1}^{*}\left(t\right)=u_{E1}^{*}\left(0|\tau_{k}\right),for\;t\in \left[\tau_{k},\tau_{k+1}\right),k=0,\;1,2,\ldots$$

The superiority of the proposed EfiMPC1 is that the subordinate objective $J_{N}\left(x_{k}\right)=L_{E1}$ representing global process performance can itself be optimized online by different PA. For each specific $PA^{j}$, there is a corresponding subordinate objective $J_{N}^{j}\left(x_{k}\right)=L_{E1}^{j}$, and the best subordinate objective $L_{E1}^{*}$ is chosen to achieve the best optimization efficiency. For example, given a specific outer layer decision variable $U_{N}^{j}$, EfiMPC1 will obtain the optimal inner layer decision variable $U_{q}^{j}$, and the corresponding global process performance is $L_{E1}^{j}$; then, EfiMPC1 will update the outer layer decision variable to generate $U_{N}^{j+1}$, and the corresponding optimal inner layer decision variable $U_{q}^{j+1}$ will produce another global process performance $L_{E1}^{j+1}$. This procedure is repeated until the optimal process performance $L_{E1}^{*}$ is found, or the optimization procedure is terminated. In this contribution, a metaheuristic algorithm is used to optimize the decision variables.

#### 3.2.1. Recursive Feasibility of EfiMPC1

**Assumption** **1.** *There always exists an optimal steady-state* $\left(x_{s},u_{s}\right)$ *such that* $f\left(x_{s},u_{s},0\right)=0,x_{s}\in X,u_{s}\in U$*. This steady state is reachable within* N *steps of control inputs from the current state* $x_{k}\in \Omega_{B}\subseteq X$.

**Assumption** **2.** *Let* $\Omega_{B}$ *, shown in Figure 7, be the feasible subspace for the system controlled by EfiMPC1, which means:*

$$\Omega_{B}=\left\{x|\exists u=\{U_{N},U_{q}\},such\;that\;Eq.\left(E1\right)\;has\;a\;solution\right\}.$$

Together with Assumption 1, there must exist a corresponding terminal subspace $\Omega_{p}\subseteq \Omega_{B}$ where the optimal steady state $x_{s}$ is in the interior of $\Omega_{p}$.

Since $x_{s}$ is in the interior of $\Omega_{p}$ and the steady state operation can be seen as a special type of periodic operation, Assumptions 1 and 2 guarantee that there always exists a feasible solution of EfiMPC1 such that $x_{k+N}=x_{s}$, q=1, and $u_{q}=u_{s}$. Thus, problem (E1) is initially feasible by assumption.

Let $X_{L}=\Omega_{p}$. At sampling instant $\tau_{k}$ and the current state $x_{k}\in \Omega_{B}\subseteq X$, assume the solution of Equation (E1) is $u_{*}^{k}=\{U_{N,*}^{k},U_{q,*}^{k}\}$, where

$$U_{N,*}^{k}=\{u_{N,*}^{k}\left(0\right),\ldots ,u_{N,*}^{k}\left(N-1\right)\}$$

and

$$U_{q,*}^{k}=\left\{u_{q,*}^{k}\left(0\right),\ldots ,u_{q,*}^{k}\left(q-1\right)\right\}.$$

At the next sampling instant $\tau_{k+1}$, there must exist a feasible solution $u_{f}^{k+1}=(U_{N,f}^{k+1},U_{q,f}^{k+1})$ for Equation (E1) such that

$$U_{N,f}^{k+1}=\{u_{N,*}^{k}\left(1\right),\ldots ,u_{N,*}^{k}\left(N-1\right),u_{q,*}^{k}\left(0\right)\}$$

and

$$U_{q,f}^{k+1}=\left\{u_{q,*}^{k}\left(1\right),\ldots ,u_{q,*}^{k}\left(q-1\right),u_{q,*}^{k}\left(0\right)\right\}.$$

Since feasibility at $\tau_{k}$ guarantees the feasibility at $\tau_{k+1}$, and by backward recursion and induction, EfiMPC1 is recursively feasible if it is initially feasible.

#### 3.2.2. Closed-Loop Stability of EfiMPC1

Based on recursive feasibility, $x(\tau_{k})$ has feasible control sequences if the problem is initially feasible, and the optimal control sequence will generate open-loop optimal periodic operation $X_{q,*}^{k}=\{x_{q,*}^{k}\left(0\right),x_{q,*}^{k}\left(1\right),\ldots ,x_{q,*}^{k}(q-1)\}$ obtained by $L_{E1}^{*}(x_{k})$.

Although the closed-loop operation may not be the periodic operation controlled by EfiMPC1, the closed-loop system is guaranteed to be bounded within the feasible subspace $\Omega_{B}$ in Figure 7. For example, at sampling instant $\tau_{k}$, the optimal control $u_{*}^{k}$ will steer the state to $x(\tau_{k+1})$ at $\tau_{k+1}$. Since $x(\tau_{k+1})$ is still feasible by recursive feasibility discussed before, it must be within the region of $\Omega_{B}$. By backward recursion and induction, once $x(\tau_{k})\in \Omega_{B}$, $x(t)\in \Omega_{B}$ for all $t>\tau_{k}$, thus, the system controlled by EfiMPC1 is bounded within the feasible subspace $\Omega_{B}$ as illustrated in Figure 7.

**Definition** **3.** *If there exists a terminal subspace* $\Omega_{p}$ *that guarantees the recursive feasibility of EfiMPC1 from Equation (E1), there must exist a corresponding feasible subspace *$\Omega_{B}$ *satisfying Assumptions 1 and 2 that bounds the closed-loop system. The states* $x(\tau_{k})\in \Omega_{B}$ *are called recursively bounded stable if and only if*

$$\left(\tau_{k}\right)\in \Omega_{B}\Rightarrow x\left(t\right)\in \Omega_{B},\forall t\geq \tau_{k},$$

*and this feasible subspace* $\Omega_{B}$ *is denoted as efficiency stability region*.

By Definition 3, once the current state satisfies $x(\tau_{k})\in \Omega_{B}$, the recursive feasibility and closed-loop stability are guaranteed, and the closed-loop trajectory is recursively bounded stable within the efficiency stability region $\Omega_{B}$.

### 3.3. Zone Control-Based Optimization Perspective

The Periodic Approximation technique can help optimize the global process performance within the inner layer of EfiMPC1, but this periodic operation imposes a stringent condition on the controlled plant. Solving this equality constraint is challenging for an online optimization solver, particularly since the controlled plant may lack any periodic operation mode. To alleviate the stringent condition imposed by periodic approximation, we introduce a zone control-based optimization approach.

The role of the terminal truncation term in an efficiency-oriented MPC can be seen as balancing two aspects: (1) economic-oriented performance, and (2) control-oriented performance. This leads to a prioritization issue: should economic-oriented performance take precedence, or should control-oriented performance?

Taking TMPC as an example, control performance is paramount, and the controller forces the system into a steady state; economic performance is a consequence of the control performance. Regarding equality constraint- and region constraint-based EMPC, control performance remains paramount, with economic performance taking a secondary role; the optimization efficiency for the economic performance within this constrained set is typically low.

In contrast, the periodic approximation technique sacrifices control performance to further enhance optimization efficiency: it optimizes terminal process performance within a terminal subspace. This parallels the concept of zone control, where the system is first controlled into a specific zone region, and then the other optimization objective is considered within that region [46,47]. Periodic approximation is implemented within a terminal region $\Omega_{p}$, which serves as a zone control region, and the global process performance is then optimized within this region.

From this perspective, periodic approximation can be viewed as a zone control-based optimization within the second-order prediction horizon q at the inner layer: the terminal state $x(t_{k+N})$ is controlled into a terminal subspace $\Omega_{p}$ within the first-order prediction horizon $H_{p}$, and the terminal truncation term is then optimized within this subspace. A Larger $\Omega_{p}$ has the potential to achieve better global process performance.

Given that steady-state operation is generally more acceptable to users, and arbitrary dynamic operations are often perceived as risky by practitioners, it is desirable to define the terminal subspace $\Omega_{p}$ as a function of the optimal steady-state $x_{s}$ satisfying $\Omega_{p}=B_{\delta }(x_{s})$, where $B_{\delta }\left(x_{s}\right)=\left\{x|{\left\|x-x_{s}\right\|}_{2}\leq \delta ,\delta >0\right\}$.

From the perspective of zone control-based optimization, one goal of an efficiency-oriented MPC is to relax control performance constraints into a larger zone rather than rigid setpoints, thereby enabling improved optimization efficiency and, consequently, better global process performance.

Periodic approximation represents a stringent optimization objective within the zone, which can be generalized to a relaxed-periodic approximation (r-PA). In r-PA, the constraint of the closed-periodic approximation defined by $x_{q}\left(q\right)=x_{q}\left(0\right)$ is replaced by a penalty parameter $p_{c}={e^{\left\|x_{q}\left(q\right)-x_{q}\left(0\right)\right\|}}^{2}\geq 1$. By multiplying PA by $p_{c}$ and removing the equality constraint $x_{q}\left(q\right)=x_{q}\left(0\right)$, the complexity of the inner layer optimization is reduced. However, this relaxation of the terminal truncation term may weaken the link between the local process performance and the global process performance.

### 3.4. Efficiency-Oriented MPC Type II

Denote the efficiency-oriented MPC based on the perspectives of relaxed-periodic approximation and the optimal steady-state zone $B_{\delta }\left(x_{s}\right)$ as Efficiency-oriented MPC Type II (EfiMPC2), and its definition is as follows:

(E2) $$L_{E2}^{*}=\underset{U_{N},U_{q}}{\min}V_{lpp}^{E2}(x\left(t_{k}\right),U_{N})+V_{ter}^{E2}(x\left(t_{k+N}\right),U_{q})$$

$$s.t.V_{lpp}^{E2}=\frac{1}{N}\int_{t_{k}}^{t_{k+N}}l_{e}\left(x\left(t\right),u\left(t\right)\right)dt$$

$$V_{ter}^{E2}=PA\left(x\left(t_{k+N}\right),u_{q}^{*}\right)$$

$$x\left(t_{k}\right)=x_{m}\left(\tau_{k}\right),\Delta t_{k}=t_{k+1}-t_{k}=\Delta t_{samp}$$

$$\dot{x}\left(t\right)=f\left(x\left(t\right),u\left(t\right),0\right),t_{k}\leq t\leq t_{k+N}$$

$$x\left(t\right)\in X_{U}\subseteq R^{n},\;u\left(t\right)\in U_{U}\subseteq R^{m},t_{k}\leq t\leq t_{k+N}$$

$$U_{N}=\left\{u_{N}\left(t_{k}\right),\ldots ,u_{N}\left(t_{k+N-1}\right)\right\}$$

$$u\left(t\right)=u_{N}\left(t_{k+i}\right),t_{k+i}\leq t<t_{k+i+1},i=0,\ldots ,N-1$$

$$U_{q}=u_{q}^{*}=\left\{u_{q}^{*}\left(0\right),\ldots ,u_{q}^{*}\left(q-1\right)\right\}$$

$$u_{q}^{*}=\underset{u_{q}}{\mathrm{argmin}}PA\left(x\left(t_{k+N}\right),u_{q}\right)$$

$$s.t.\;PA\left(x\left(t_{k+N}\right),u_{q}\right)=\frac{\sum_{j=0}^{q-1}l_{e}\left(x_{q}\left(j\right),u_{q}\left(j\right)\right)}{q}\cdot p_{c}$$

$$x_{q}\left(0\right)=x\left(t_{k+N}\right),p_{c}=e^{{\left\|x_{q}\left(q\right)-x_{q}\left(0\right)\right\|}^{2}}$$

$$x_{q}\left(j+1\right)=f_{d}\left(x_{q}\left(j\right),u_{q}\left(j\right),0\right),j=0,\;1,\ldots ,q-1$$

$$x_{q}\left(j\right)\in X_{L}=B_{\delta }\left(x_{s}\right)\subseteq R^{n},u_{q}\left(j\right)\in U_{L}\subseteq R^{n},j=0,\;1,\ldots ,q-1$$

Unlike EfiMPC1, EfiMPC2 normalizes $V_{lpp}^{E2}$ and $V_{ter}^{E2}$, constrains the terminal state $x\left(t_{k+N}\right)$ into $B_{\delta }(x_{s})$ at the outer layer, and replaces the equality constraint of the periodic $x_{q}\left(q\right)=x_{q}\left(0\right)$ with a penalty parameter $p_{c}$ at the inner layer, where ${\left\|x_{q}\left(q\right)-x_{q}\left(0\right)\right\|}^{2}={\left(\sqrt{x_{q}\left(q\right)-x_{q}\left(0\right)}\right)}^{2}$. Consequently, EfiMPC2 defined by Equation (E2) is easier to handle online than EfiMPC1, as it does not require solving the strict equality constraint. In EfiMPC1, $X_{L}=X_{U}$ is used by default, which implies $B_{\delta }\left(x_{s}\right)=X_{U}$, and this larger space gives EfiMPC1 the potential to achieve better optimization efficiency than EfiMPC2. The main differences between EfiMPC1 and EfiMPC2 are the inner optimization problems in Equations (E1) and (E2).

## 4. A CSTR Case Study

### 4.1. Application to a Chemical Process Example

This section demonstrates the effectiveness of the proposed controllers, EfiMPC1 and EfiMPC2, using a chemical process example from [48]. Metaheuristic algorithms serve as the embedded optimization solvers. Researchers have proposed various metaheuristics, including Particle Swarm Optimization (PSO) [49], Salp Swarm Algorithm (SSA) [50], Grey Wolf Optimizer (GWO) [51], Integrated Optimization Algorithm (IOA) [52], and Optimal Stochastic Process Optimizer (OSPO) [53]. In this contribution, we use GWO as the optimization solver. The simulation results for EfiMPC1 and EfiMPC2 are compared with those of traditional tracking MPC (TMPC), equality-constraint based Economic MPC (Equ-EMPC), and region-constraint based Economic MPC (Reg-EMPC) strategies.

The chemical process considered here is the oxidation of ethylene to ethylene oxide in a non-isothermal continuously stirred tank reactor (CSTR). The process is modeled through three complex reactions:

(4) $$\begin{array}{c} C_{2}H_{4}+\frac{1}{2}O_{2}\to C_{2}H_{4}O\\ C_{2}H_{4}+3O_{2}\to 2CO_{2}+2H_{2}O\\ C_{2}H_{4}O+\frac{5}{2}O_{2}\to 2CO_{2}+2H_{2}O \end{array}$$

In [54], the dimensionless material and energy balances for the CSTR were developed using the reactions described in Equation (4) from [55]. The resulting dimensionless equations representing the reactor dynamics are given by $\dot{x}=f(x,u,0)$, defined as follows:

(5) $$\begin{array}{c} {\dot{x}}_{1}=u_{1}\left(1-x_{1}x_{4}\right)\;\\ {\dot{x}}_{2}=u_{1}\left(u_{2}-x_{2}x_{4}\right)-A_{1}e^{\gamma_{1}/x_{4}}{\left(x_{2}x_{4}\right)}^{0.5}-A_{2}e^{\gamma_{2}/x_{4}}{\left(x_{2}x_{4}\right)}^{0.25}\\ {\dot{x}}_{3}=-u_{1}x_{3}x_{4}+A_{1}e^{\gamma_{1}/x_{4}}{\left(x_{2}x_{4}\right)}^{0.5}-A_{3}e^{\gamma_{3}/x_{4}}{\left(x_{2}x_{4}\right)}^{0.5}\\ {\dot{x}}_{4}=\frac{u_{1}}{x_{1}}\left(1-x_{4}\right)+\frac{B_{1}}{x_{1}}e^{\gamma_{1}/x_{4}}{\left(x_{2}x_{4}\right)}^{0.5}+\frac{B_{2}}{x_{1}}e^{\gamma_{2}/x_{4}}{\left(x_{2}x_{4}\right)}^{0.25}+\frac{B_{3}}{x_{1}}e^{\gamma_{3}/x_{4}}{\left(x_{2}x_{4}\right)}^{0.5}-\frac{B_{4}}{x_{1}}(x_{4}-T_{c}) \end{array}$$

where the dimensionless state variables $x_{1},x_{2},x_{3}$, and $x_{4}$ represent the dimensionless gas density, ethylene concentration, ethylene oxide concentration, and temperature in the reactor, respectively. $u_{1},u_{2}$ denote the feed volumetric flow rate and the concentration of ethylene in the feed, respectively. The parameters in Equation (5) are defined in [48].

The objective of the control strategy in this CSTR application is to optimize the global process performance (profit) $P(t_{0},t_{f})$ over the entire process operation from $t_{0}$ to $t_{f}$, which can be formulated as a minimization problem as follows:

(6) $$\underset{u}{\max}P\left(t_{0},t_{f}\right)=-\underset{u}{\min}P\left(t_{0},t_{f}\right)=\underset{u}{\min}\int_{t_{0}}^{t_{f}}-l_{e}\left(\tau \right)d\tau =\underset{u}{\min}J_{\infty }\left(x(t_{0})\right)$$

$$s.t.C_{r}=\int_{t_{0}}^{t_{f}}u_{1}\left(\tau \right)u_{2}\left(\tau \right)d\tau \leq C_{fs}$$

where $J_{\infty }\left(x(t_{0})\right)$ is the superordinate objective representing the global objective; $t_{0}$ and $t_{f}$ are the initial and the final time instants of the chemical process operation, respectively; $C_{r}$ represents the amount of the reactant feedstock during the process, which is limited by the maximum inventory constant $C_{fs}$; $l_{e}\left(\tau \right)$ is the instantaneous process profit of interest representing the yield of oxide, and it is defined as follows [48]:

(7) $$l_{e}\left(t\right)=l_{e}^{CSTR}\left(x(t),u(t)\right)=u_{1}\left(t\right)x_{3}\left(t\right)x_{4}\left(t\right)$$

Due to the actuator limitations, constraints on u should satisfy $0.0704\leq u_{1}\leq 0.7042$, $0.2465\leq u_{2}\leq 2.4648$, $-0.1\leq {\Delta u}_{1}\leq 0.1$, and $-0.3\leq {\Delta u}_{2}\leq 0.3$. The reactor is initialized at $x_{0}=$ [0.99, 1.5, 0.3, 1.0], and a sampling period for the process $\Delta t_{sam}=1s$ is used. The first order Runge-Kutta numerical integration method is used to obtain the discrete model $f_{d}$ from Equation (5), and the integration step is $h_{u}=10^{-2}$ s. The limited reactant feedstock constraint is defined as $C_{fs}=0.175\left(t_{f}-t_{0}\right)$. $x_{s}$ = [0.9956, 1.7511, 0.2511, 1.0043] is the optimal steady state, and $u_{s}=$[0.0704, 2.4648] is the corresponding optimal input vector.

The global optimization problem (6) is impractical due to its large optimization horizon, and a receding horizon strategy is employed to solve this problem. Therefore, the goal of the control strategy at the current time instant $t_{k}$ is to approximate the superordinate objective $J_{\infty }\left(x(t_{k})\right)$ with the optimal subordinate objective $J_{N}^{*}\left(x(t_{k})\right)$, whose prediction horizon N is much smaller than the remaining horizon $\left(t_{f}-t_{k}\right)$.

The EfiMPC1 and EfiMPC2 control strategies for this CSTR application are defined in Equation (8) and Equation (9), respectively, as follows:

(8) $$\mathrm{EfiMPC}1:\;L_{Efi1}^{*}=\underset{U_{N},U_{q}}{\min}-\left(V_{lpp}^{E1}\left(x\left(t_{k}\right),U_{N}\right)+V_{ter}^{E1}\left(x\left(t_{k+N}\right),U_{q}\right)\right)$$

$$s.t.V_{lpp}^{E1}=\int_{t_{k}}^{t_{k+N}}u_{1}\left(t\right)x_{3}\left(t\right)x_{4}\left(t\right)dt$$

$$V_{ter}^{E1}=PA\left(x\left(t_{k+N}\right),u_{q}^{*}\right)\cdot \left(t_{f}-t_{k+N}\right)\;$$

(8-c1) $$Equation\;\left(5\right),t_{k}\leq t\leq t_{k+N}\;\;$$

(8-c2) $$x\left(t_{k}\right)=x_{m}\left(\tau_{k}\right),\Delta t_{k}=t_{k+1}-t_{k}=\Delta t_{samp}$$

(8-c3) $$0.0704\leq u_{1}\left(t\right)\leq 0.7042,0.2465\leq u_{2}\left(t\right)\leq 2.4648,t_{k}\leq t\leq t_{k+N}$$

(8-c4) $$-0.1\leq {\Delta u}_{1}\leq 0.1,-0.3\leq {\Delta u}_{2}\leq 0.3,t_{k}\leq t\leq t_{k+N}$$

(8-c5) $$\int_{t_{k}}^{t_{k+N}}u_{1}\left(t\right)u_{2}\left(t\right)dt\leq 0.175\left(t_{k+N}-t_{k}\right)$$

$$U_{N}=\left\{u_{N}\left(t_{k}\right),\ldots ,u_{N}\left(t_{k+N-1}\right)\right\}$$

$$u\left(t\right)=u_{N}\left(t_{k+i}\right),t_{k+i}\leq t<t_{k+i+1},i=0,\ldots ,N-1\;$$

$$U_{q}=u_{q}^{*}=\left\{u_{q}^{*}\left(0\right),\ldots ,u_{q}^{*}\left(q-1\right)\right\}$$

$$u_{q}^{*}=\underset{u_{q}}{\mathrm{argmin}}PA\left(x\left(t_{k+N}\right),u_{q}\right)$$

$$s.t.\;PA\left(x\left(t_{k+N}\right),u_{q}\right)=\frac{\sum_{j=0}^{q-1}u_{q,1}\left(j\right)x_{q,3}\left(j\right)x_{q,4}\left(j\right)}{q}$$

$$x_{q}\left(0\right)=x\left(t_{k+N}\right),x_{q}\left(q\right)=x_{q}\left(0\right)$$

$$x_{q}\left(j+1\right)=f_{d}^{CSTR}\left(x_{q}\left(j\right),u_{q}\left(j\right),0\right),j=0,\;1,\ldots ,q-1$$

$$0.0704\leq u_{q,1}\left(j\right)\leq 0.7042,\;0.2465\leq u_{q,2}\left(j\right)\leq 2.4648,j=0,\;1,\ldots ,q-1$$

$$\sum_{j=0}^{q-1}u_{q,1}\left(j\right)u_{q,2}\left(j\right)\leq 0.175\cdot q$$

where $f_{d}^{CSTR}$ is the discrete version of Equation (5). The definitions in Equation (8) correspond to those in Equation (E1), and the parameters N=10, q=10 are used.

(9) $$\mathrm{EfiMPC}2:\;L_{Efi2}^{*}=\underset{U_{N},U_{q}}{\min}-\left(V_{lpp}^{E2}\left(x\left(t_{k}\right),U_{N}\right)+V_{ter}^{E2}\left(x\left(t_{k+N}\right),U_{q}\right)\right)$$

$$s.t.V_{lpp}^{E2}=\frac{1}{N}\int_{t_{k}}^{t_{k+N}}u_{1}\left(t\right)x_{3}\left(t\right)x_{4}\left(t\right)dt$$

$$V_{ter}^{E1}=PA\left(x\left(t_{k+N}\right),u_{q}^{*}\right)$$

(8-c1) $$Equation\;\left(5\right),t_{k}\leq t\leq t_{k+N}\;\;$$

(8-c2) $$x\left(t_{k}\right)=x_{m}\left(\tau_{k}\right),\Delta t_{k}=t_{k+1}-t_{k}=\Delta t_{samp}$$

(8-c3) $$0.0704\leq u_{1}\left(t\right)\leq 0.7042,0.2465\leq u_{2}\left(t\right)\leq 2.4648,t_{k}\leq t\leq t_{k+N}$$

(8-c4) $$-0.1\leq {\Delta u}_{1}\leq 0.1,-0.3\leq {\Delta u}_{2}\leq 0.3,t_{k}\leq t\leq t_{k+N}$$

(8-c5) $$\int_{t_{k}}^{t_{k+N}}u_{1}\left(t\right)u_{2}\left(t\right)dt\leq 0.175\left(t_{k+N}-t_{k}\right)$$

$$U_{N}=\left\{u_{N}\left(t_{k}\right),\ldots ,u_{N}\left(t_{k+N-1}\right)\right\}$$

$$u\left(t\right)=u_{N}\left(t_{k+i}\right),t_{k+i}\leq t<t_{k+i+1},i=0,\ldots ,N-1\;$$

$$U_{q}=u_{q}^{*}=\left\{u_{q}^{*}\left(0\right),\ldots ,u_{q}^{*}\left(q-1\right)\right\}$$

$$u_{q}^{*}=\underset{u_{q}}{\mathrm{argmin}}PA\left(x\left(t_{k+N}\right),u_{q}\right)$$

$$s.t.\;PA\left(x\left(t_{k+N}\right),u_{q}\right)=\frac{\sum_{j=0}^{q-1}u_{q,1}\left(t\right)x_{q,3}\left(t\right)x_{q,4}\left(t\right)}{q}\cdot p_{c}$$

$$x_{q}\left(0\right)=x\left(t_{k+N}\right),p_{c}=e^{{\left\|x_{q}\left(q\right)-x_{q}\left(0\right)\right\|}^{2}}$$

$$x_{q}\left(j+1\right)=f_{d}^{CSTR}\left(x_{q}\left(j\right),u_{q}\left(j\right),0\right),j=0,\;1,\ldots ,q-1$$

$$0.0704\leq u_{q,1}\left(j\right)\leq 0.7042,\;0.2465\leq u_{q,2}\left(j\right)\leq 2.4648,j=0,\;1,\ldots ,q-1$$

$$x_{q}\left(j\right)\in X_{L}=B_{\delta }\left(x_{s}\right),j=0,\;1,\ldots ,q-1$$

$$\sum_{j=0}^{q-1}u_{q,1}\left(j\right)u_{q,2}\left(j\right)\leq 0.175\cdot q$$

where the definitions of the parameters are the same as those in Equation (8), except that $B_{\delta }\left(x_{s}\right)=\left\{x|{\left\|x_{q}\left(j\right)-x_{s}\right\|}^{2}\leq \delta_{Efi2}\right\}$.

The comparative control strategies TMPC, Equ-EMPC, and Reg-EMPC are defined as follows:

(10) $$\mathrm{TMPC}:\;L_{TMPC}^{*}=\underset{u}{min}\int_{t_{k}}^{t_{k+N^{\prime }}}{\left\|x\left(t\right)-x_{s}\right\|}_{Q}^{2}+{\left\|u\left(t\right)-u_{s}\right\|}_{R}^{2}dt\;$$

s.t. Equations(8-c1)~(8-c5)

(11) $$\mathrm{Equ}-\mathrm{EMPC}:\;L_{Equ-EMPC}^{*}=\underset{u}{min}\int_{t_{k}}^{t_{k+N^{\prime }}}-u_{1}\left(t\right)x_{3}\left(t\right)x_{4}\left(t\right)dt$$

s.t. Equations(8-c1)~(8-c5)

$$x\left(t_{k+N}\right)-x_{s}=0$$

(12) $$\mathrm{Reg}-\mathrm{EMPC}:\;L_{Reg-EMPC}^{*}=\underset{u}{min}\int_{t_{k}}^{t_{k+N^{\prime }}}-u_{1}\left(t\right)x_{3}\left(t\right)x_{4}\left(t\right)dt$$

s.t. Equations(8-c1)~(8-c5)

$${\left\|x\left(t_{k+N}\right)-x_{s}\right\|}_{Q}^{2}\leq \delta_{R}$$

where Q and R are positive definite matrices, and $\delta_{R}$ is a non-negative scalar value.

### 4.2. Simulation Results

The parameters of the GWO solver embedded in efficiency-oriented controllers are the default values defined in [56]. The parameters in Equation (8) through Equation (12) are reported in Table 1. The prediction horizons for TMPC, Equ-EMPC, and Reg-EMPC are set equal to $N^{\prime }$ for simplicity, and $x_{s}$ is the optimal steady state.

The ideal optimal global process performance of the current CSTR application from $x_{0}$ is $J_{\infty }^{*}\left(x_{0}\right)=-1.0678$ (minimum is the optimum), and this value is utilized to compute the optimization efficiency defined in Equation (1). The closed-loop process performances obtained by the comparative control strategies are $J_{N}^{i}\left(x_{0}\right)$, and a larger value of optimization efficiency, imply greater superiority of the corresponding control strategy.

The closed-loop process performance and the optimization efficiency under the five comparative control strategies for the CSTR application are reported in Table 2. The results show that the proposed EfiMPC1 exhibits the best closed-loop process performance at −1.010810535 (minimum is the optimum), and EfiMPC2 has a closed-loop process performance at −0.983458174, taking second place. The closed-loop process performance of EfiMPC1 exhibits improvements of 11.4%, 9.5%, and 7.3% compared with TMPC, Equ-EMPC, and Reg-EMPC strategies, respectively. These results demonstrate that optimization efficiency can indicate the superiority of the comparative control strategies as the best control strategy, and EfiMPC1 has the highest optimization efficiency, while the less effective strategies have lower values. Thus, the effectiveness of the optimization efficiency has also been demonstrated.

**Figure 8:**
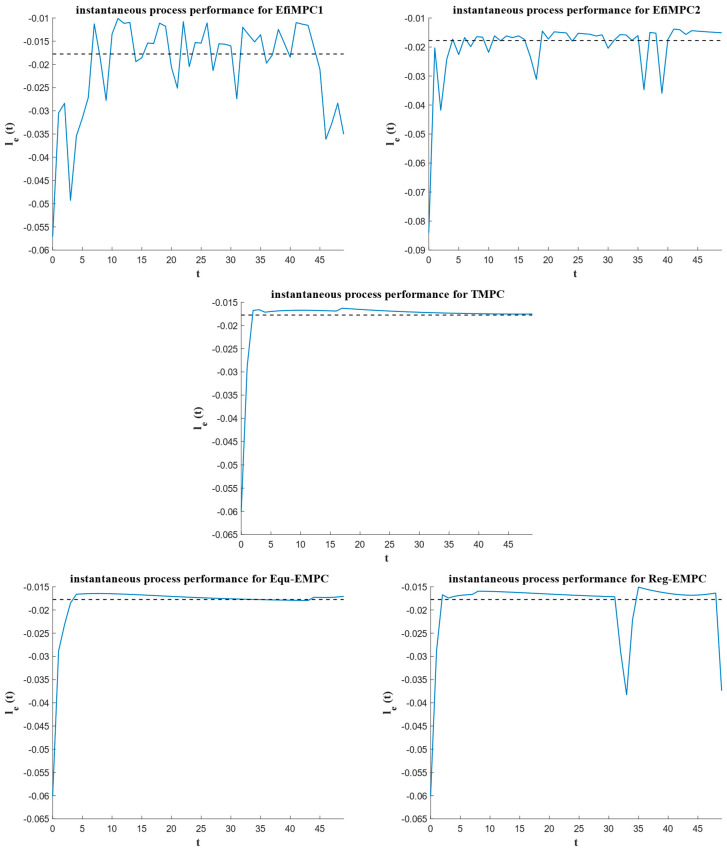
The instantaneous process performance of the comparative control strategies. (The doted lines indicate the performance of optimal steady-state operation).

**Figure 9:**
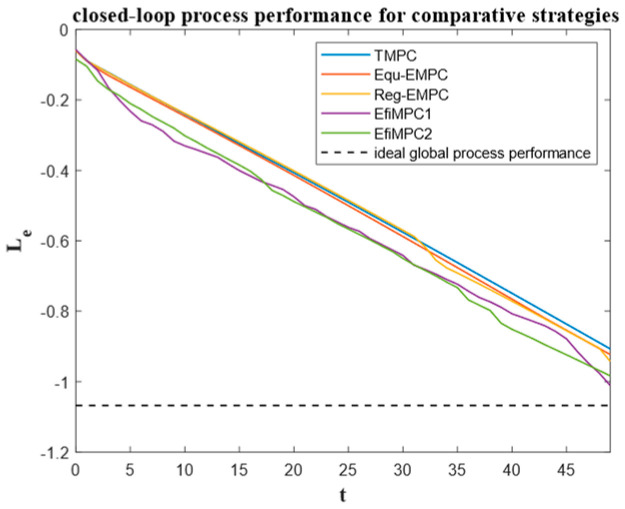
The closed-loop process performance of the comparative control strategies.

**Figure 10:**
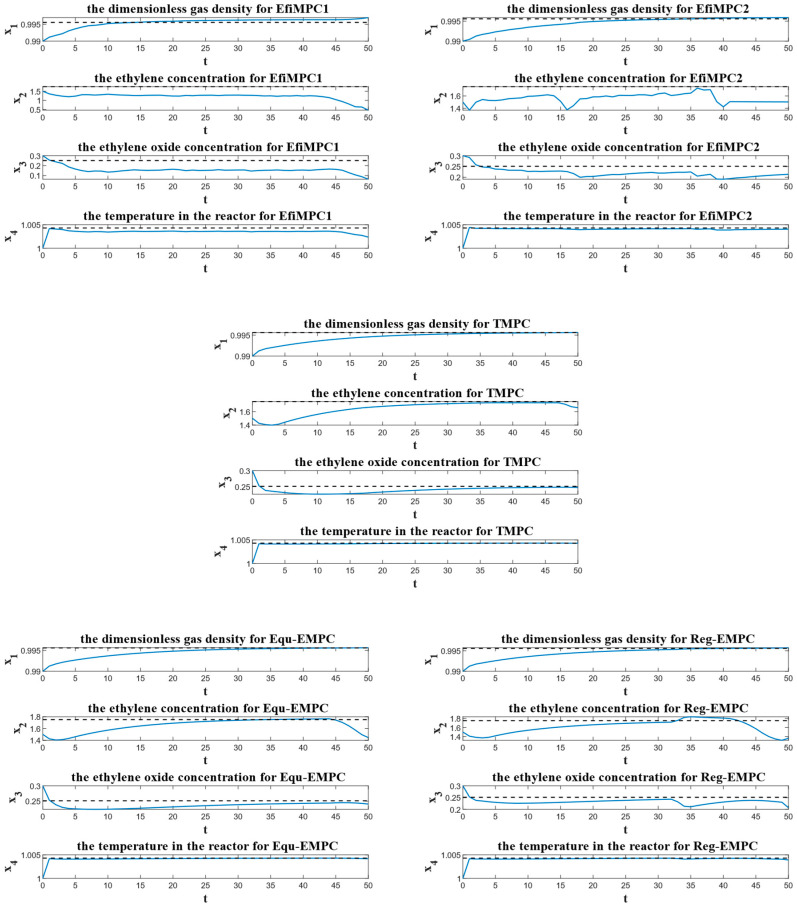
The state trajectories of the comparative control strategies. (The doted lines indicate optimal steady states).

**Figure 11:**
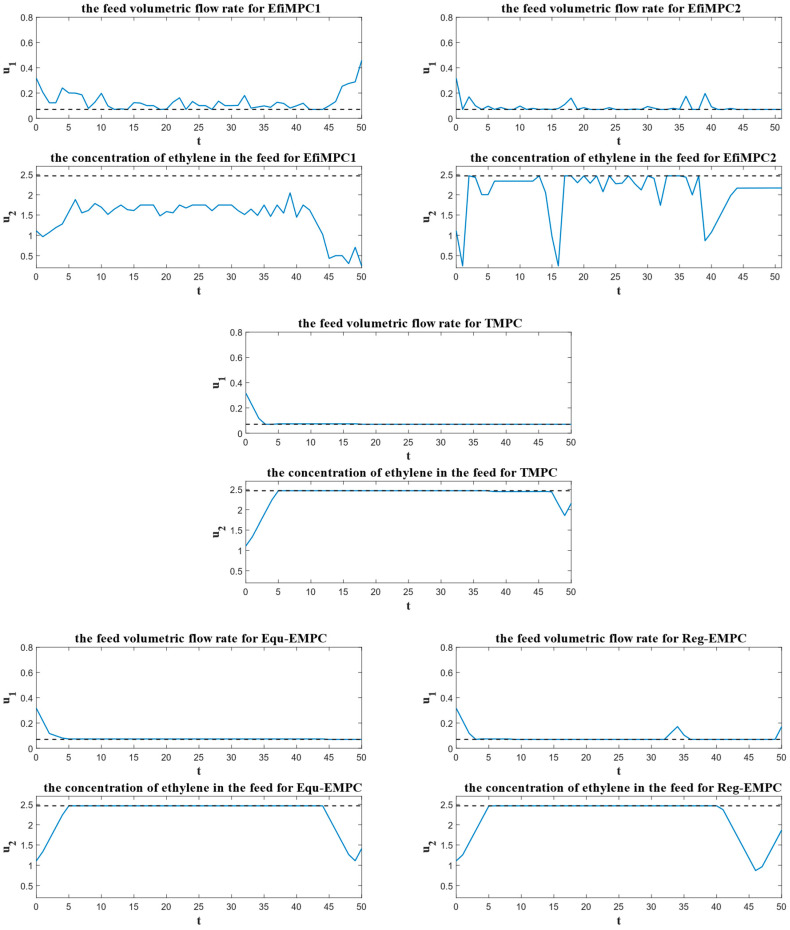
The input trajectories of the comparative control strategies. (The dotted lines indicate the optimal steady state control inputs).

To further investigate the performance of the comparative control strategies, the instantaneous process performances over the sample instants are depicted in Figure 8, the closed-loop process performances are depicted in Figure 9, and the corresponding state trajectories and input trajectories are depicted in Figure 10 and Figure 11, respectively.

The closed-loop process performances are illustrated in Figure 9. It is evident that efficiency-oriented MPC strategies EfiMPC1 and EfiMPC2 exhibit better closed-loop process performance than that of the TMPC, Equ-EMPC, and Reg-EMPC strategies. The closed-loop process performance values of EfiMP1 and EfiMPC2 are closer to the ideal global process performance $L_{e}^{o}=-1.0678$, marked by the dashed line. The slopes of the performance trajectories controlled by TMPC, Equ-EMPC, and Reg-EMPC are relatively flat, whereas the slopes of EfiMPC1 and EfiMPC2 are more dynamic. This occurs because EfiMPC1 and EfiMPC2 can optimize the subordinate objective to achieve better global process performance online.

EfiMPC1 has the best closed-loop process performance (−1.010810535), demonstrating the effectiveness of the efficiency-oriented MPC for optimizing the global process performance. The closed-loop process performance for EfiMPC2 is −0.983458174, taking the second place, indicating that the link between local process performance and global process performance is weaker than that of EfiMPC1.

The reason for the superiority of the efficiency-oriented MPC can be partially explained by Figure 10 and Figure 11. As shown in Figure 10, the dashed lines represent the optimal steady state $x_{s}$, and TMPC, Equ-EMPC, and Reg-EMPC steer their states into close proximity to this $x_{s}$. In contrast, EfiMPC1 and EfiMPC2 do not steer their states into close proximity to this optimal steady state; instead, their state trajectories behave dynamically to find a better global process performance. Additionally, the dynamic range of EfiMPC1 is larger than that of EfiMPC2 because EfiMPC2 restricts the system states to a pre-determined zone region.

Figure 11 illustrates the input trajectories of the comparative control strategies. The dashed lines represent the corresponding optimal steady state inputs $u_{s}$, and the control inputs of TMPC, Equ-EMPC, and Reg-EMPC fall into a small neighborhood of $u_{s}$ for most of the operation time. For EfiMPC1 and EfiMPC2, however, their control inputs are more dynamic. These dynamic control inputs are the result of the online optimization for the global process performance. The control inputs of EfiMPC1 are more dynamic than those of EfiMPC2, which contributes to the better closed-loop process performance of EfiMPC1 compared to EfiMPC2.

## 5. Discussion

The simulation results reported in Figure 8, Figure 9, Figure 10 and Figure 11 show that the efficiency-oriented MPC generates better global process performance, and EfiMPC1 is the best control strategy, yielding the optimal closed-loop process performance.

The reason for the efficiency-oriented MPC outperforming TMPC, Equ-EMPC, and Reg-EMPC is that it behaves dynamically, as shown in Figure 10 and Figure 11, and these dynamic behaviors result from the nested structure aiming to optimize the global process performance.

EfiMPC2 uses a zone-control-based approach, which makes its optimization efficiency lower than that of the EfiMPC1. However, EfiMPC2 has its own strengths: EfiMPC2 replaces the strict equality condition in EfiMPC1 with a penalty parameter, which reduces computational complexity and enlarges the feasible region, thus, the online optimization procedure of EfiMPC2 is easier to handle. In addition, EfiMPC2 can also be applied to applications where the controlled plant does not have a periodic operation. Therefore, for systems that have periodic operations, EfiMPC1 can be used to achieve better global process performance, and for systems for which obtaining a periodic operation is difficult, EfiMPC2 can be used to achieve better global process performance.

In this contribution, the prediction horizon of TMPC, Equ-EMPC, and Reg-EMPC is $N^{\prime }=N+q$, thus, the superiority of the efficiency-oriented MPC does not stem from modifying the length of the prediction horizon but rather from the nested optimization structure. Here, q=10 is kept constant for brevity, but q can also be regarded as a parameter to be optimized to further improve the optimization efficiency.

## 6. Conclusions

A novel control strategy, a named efficiency-oriented MPC capable of generating global process performance is proposed in this contribution. The efficiency-oriented MPC divides the global process performance into two components: local process performance and a terminal truncation term. By introducing the concept of optimization efficiency, the goal of optimizing the global process performance has been transformed into optimizing the optimization efficiency. The proposed efficiency-oriented MPC is inherently a nested optimization problem, where the outer layer optimization problem concerns the local process performance, and the inner layer optimization problem concerns the terminal truncation term. This nested structure renders the efficiency-oriented MPC an intelligent control strategy, thus making the subordinate objective optimized to better approximate the superordinate objective, and thereby enabling direct online optimization of the global process performance.

Periodic approximation was proposed as a specific terminal truncation technique to construct Efficiency-Oriented MPC Type I (EfiMPC1), and the recursive feasibility and the closed-loop stability of EfiMPC1 were discussed. Relaxed-periodic approximation and zone-based optimization perspectives were then discussed to construct Efficiency-Oriented MPC Type II (EfiMPC2). Both types of Efficiency-Oriented MPC were tested in a CSTR application, and they were compared with TMPC, equality-constraint-based EMPC (Equ-EMPC), and region-constraint-based EMPC (Reg-EMPC) control strategies. The simulation results demonstrated the effectiveness of the proposed efficiency-oriented MPC.

This contribution represents the initial idea of the efficiency-oriented MPC. For future studies, a dedicated optimization solver for the efficiency-oriented MPC, considering its nested structure, could be constructed. In addition, various values of the second-order prediction horizon q could be investigated. Moreover, the role of smart sensors in achieving the performance of the efficiency-oriented MPC could be directly researched.

## Figures and Tables

**Table 1:** The parameters in Equations (8)–(12).

Parameter	Value	Parameter	Value
Q	diag([1,1,1,1])	R	diag([1,1])
$t_{0}$	0 s	$t_{f}$	50 s
$\Delta t_{samp}$	1 s	$\delta_{Efi2}$	0.3
$\delta_{R}$	0.3	N	10
q	10	$N^{\prime }=N+q$	20
$x_{0}$	[0.99, 1.5, 0.3, 1.0]	$x_{s}$	[0.9956, 1.7511, 0.2511, 1.0043]

**Table 2:** Simulation Results for CSTR under different control strategies.

Strategy	Closed-Loop Performance	Optimization Efficiency
EfiMPC1	−1.010810535	0.9446
EfiMPC2	−0.983458174	0.9191
TMPC	−0.906837645	0.8513
Equ-EMPC	−0.922916531	0.8651
Reg-EMPC	−0.942180054	0.8819

## Data Availability

Data are contained within the article.
